# Global trends and hotspots in research of paronychia: A bibliometric analysis

**DOI:** 10.1097/MD.0000000000039838

**Published:** 2024-09-27

**Authors:** Chaoxi Zhou, Guangrong Yu, Qinglei Wang, Zhaoyi Yang, Huimin Wang, Yongzhen Zhao

**Affiliations:** a Department of Emergency Surgery, Beijing Geriatric Hospital, Beijing, China; b Department of Orthopedics, Beijing Geriatric Hospital, Beijing, China.

**Keywords:** bibliometric, chronic paronychia, EGFRI, paronychia, surgery

## Abstract

**Background:**

Paronychia is a prevalent clinical disease profoundly affecting patients’ quality of life. As ongoing evolution in modern living environments, factors contributing to paronychia are becoming increasingly diverse. Therefore, a further understanding about latest trend of paronychia is imperative and pressing.

**Methods:**

A systematic literature search was performed based on Web of Science Core Collection and Science Citation Index Expanded. The search parameters encompassed keywords associated with paronychia from 1980 to 2023, and rigorous data cleaning procedures were executed to maintain the analysis’s relevance and dependability, supplemented by a thorough examination of abstracts and titles. Visibility analysis was conducted with Citespace and VOSviewer tools to explore the publication trends, collaborative networks, and impactful studies.

**Results:**

A total of 595 articles were included in this study. The annual publication trends exhibited a significant increase since 1990, reached a peak of 41 articles in 2021. Collaborative relationships among countries demonstrated strong connections, with the United States leading in both publication volume, citation records and international cooperation. Keyword analysis indicated that in recent years, a substantial body of research has concentrated on paronychia issues caused by epidermal growth factor receptor inhibitors (EGFRI)-class drugs, such as Gefitinib, Erlotinib, and Afatinib, in the context of tumor treatment.

**Conclusion:**

In this area, most of the recent hotspots are not focused on the basic research about paronychia due to the basic research about traditional paronychia already reached a relative mature stage. However, with the widespread clinical application of EGFRI anticancer drugs, the incidence of drug-induced paronychia is inevitably on the rise. Therefore, with the expanding diversity in the etiology of paronychia, this area deserves a multiple discipline cooperation with a much wider international communication.

## 1. Introduction

Paronychia, a common dermatological condition marked by infection around the nail folds, poses a significant global health concern. Untreated in a timely manner, it can escalate to abscess formation, intensifying pain and swelling,with an increased risk of systemic infections if bacteria enter the bloodstream.^[1,2]^ Severe cases may suffer systemic symptoms like fever and malaise,^[3]^ while chronic paronychia can cause lasting changes in the nail fold structure, affecting both appearance and function.^[4,5]^

In recent years, the incidence of paronychia has exhibited some new trends. With the improvement of personal hygiene awareness, people are increasingly prioritizing the cleanliness and care of their hands and feet, which may theoretically contributes to a reduced incidence of paronychia. However, the shifts in modern lifestyles also bring forth new challenges. Daily habits like frequent exposure to detergents, chemicals and hairdressing can potentially damage the skin’s natural barrier, thus elevating the risk of developing paronychia.^[6–8]^ Moreover, with the ongoing advancements in clinical pharmacology, numerous medications such as Indinavir, Gefitinib, Afatinib, etc., while effectively treating primary diseases, are also causing an uptick in paronychia cases due to their side effects.^[9–13]^ These newly emerged pathogenic factors undoubtedly pose new problems and challenges for the clinical diagnosis and treatment of paronychia.

As the prevalence of paronychia continues, bibliometric software emerges as a vital tool for exploring the research trends, perfornance relationships, and hotspots in a specific domain.^[14–17]^ Yet so far, a systematic and in-depth bibliometric analysis of paronychia remains lacking. Therefore, by applying bibliometric analysis to paronychia related publications, we aim to uncover the research status and frontiers in this dynamic field.

## 2. Methods

### 2.1. Literature search

**Figure 1.**
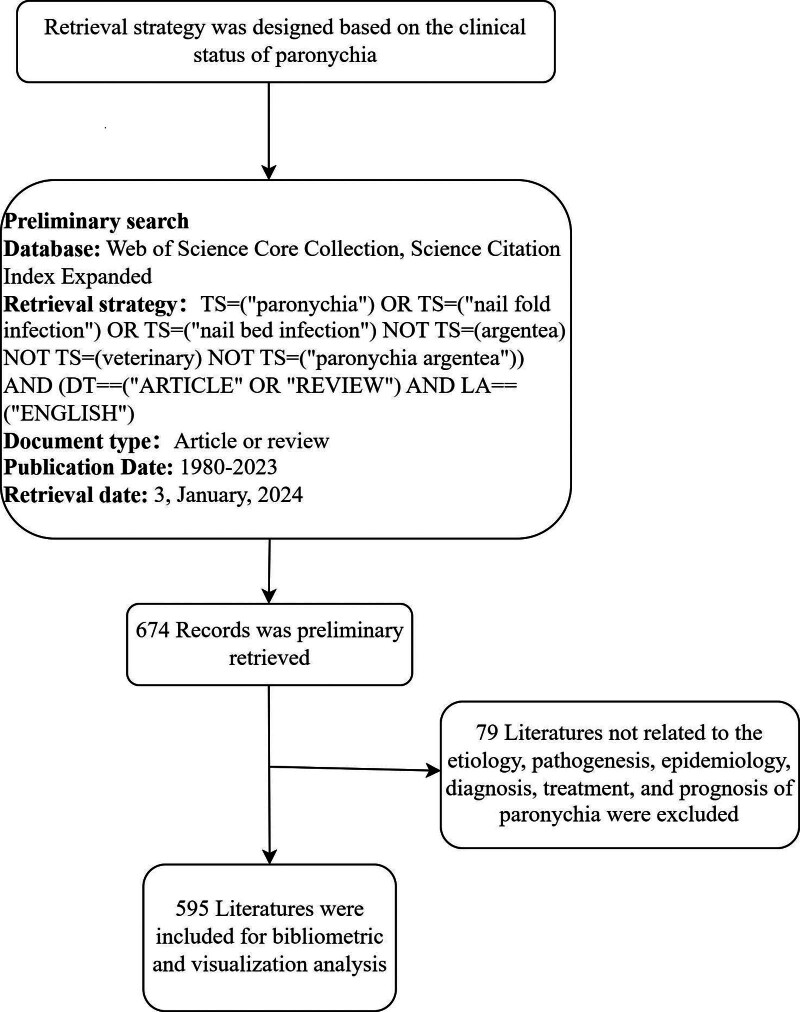
Flow diagram of literature search and screening.

Since the Science Citation Index (SCI) database includes a large number of high-quality academic journals that cover the most influential literature in our research field. Therefore, We initiated a comprehensive search across Web of Science Core Collection (WOSCC), Science Citation Index Expanded (SCI-Expanded). Following is the search formula: (TS=(“paronychia”) OR TS=(“nail fold infection”) OR TS=(“nail bed infection”) NOT TS = (argentea) NOT TS = (veterinary) NOT TS=(“paronychia argentea”)) AND (DT==(“ARTICLE” OR “REVIEW”) AND LA==(“ENGLISH”)). The retrieval date was January 3, 2024 (Fig. 1).

### 2.2. Data retrieval and analysis

Ensuring the reliability and relevance of the bibliometric analysis is crucial, and a well designed data cleaning process help establish a solid foundation for a more accurate and targeted analysis of trends in paronychia research. Therefore, 3 authors worked independently conducted a meticulous review of the literature, focusing primarily on abstracts and titles, excluding studies without relevance to the core themes of paronychia: its pathogenesis, etiology, epidemiology, treatment, and prognosis. Additionally, the retracted records were removed to ensure that each study contributes unique value to the subsequent bibliometric analysis.

After the data retrieved from WoS which include Full Record and Cited References in “plain text” format. Then all the publication data was imported into bibliometric software of Citespace, VOSviewer, and Bibliometrix for Literature Visualization Analysis in this work.

## 3. Results

### 3.1. Trend in annual publication volume and citations

**Figure 2.**
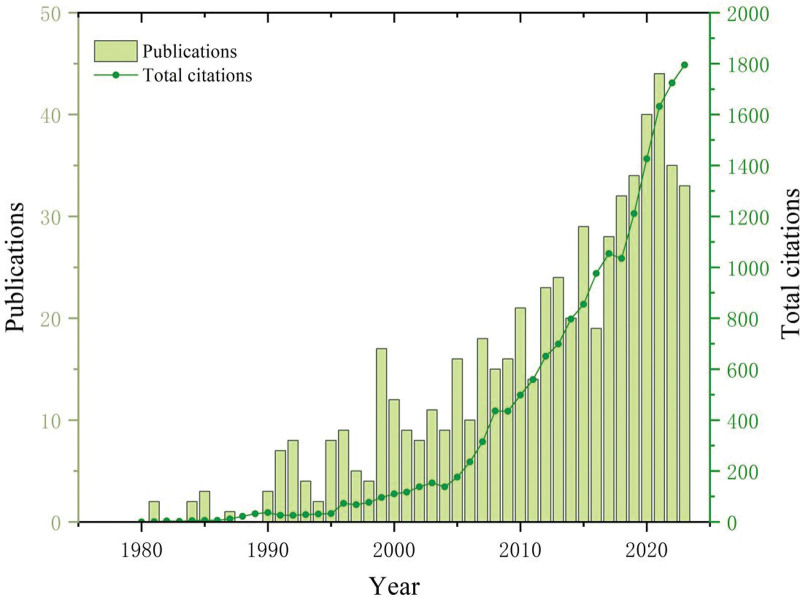
The international annual publication and citation trend of research about paronychia from 1980 to 2023.

A total of 595 articles were included in this study (see Text file, Supplemental Digital Content, http://links.lww.com/MD/N639, which demonstrates the papers included for bibliometric analysis). From 1980 to 1990, there were several years without any publications related to paronychia, and the overall output was scant (Fig. 2). However, starting from 1990, research literature about paronychia began to be consistently published annually, with the publication volume showing a fluctuating increase over time. For most of the years during the period from 1990 to 2006, the annual publication volumes usually below 10, except 1999 and 2005 exceeded 10 articles. From 2007 to 2012, the annual publication volume consistently fell within the range of 10 to 20 articles per year. Since 2013, the annual publication volume has consistently remained above 20 articles, reaching its peak of 41 articles in 2021. During 1980 to 2000, the annual total citation remained consistently below 100, exhibiting slow growth. However, after the year 2000, the total citations increased rapidly, reaching a peak of 1795 in 2023.

### 3.2. Country/region

**Figure 3.**
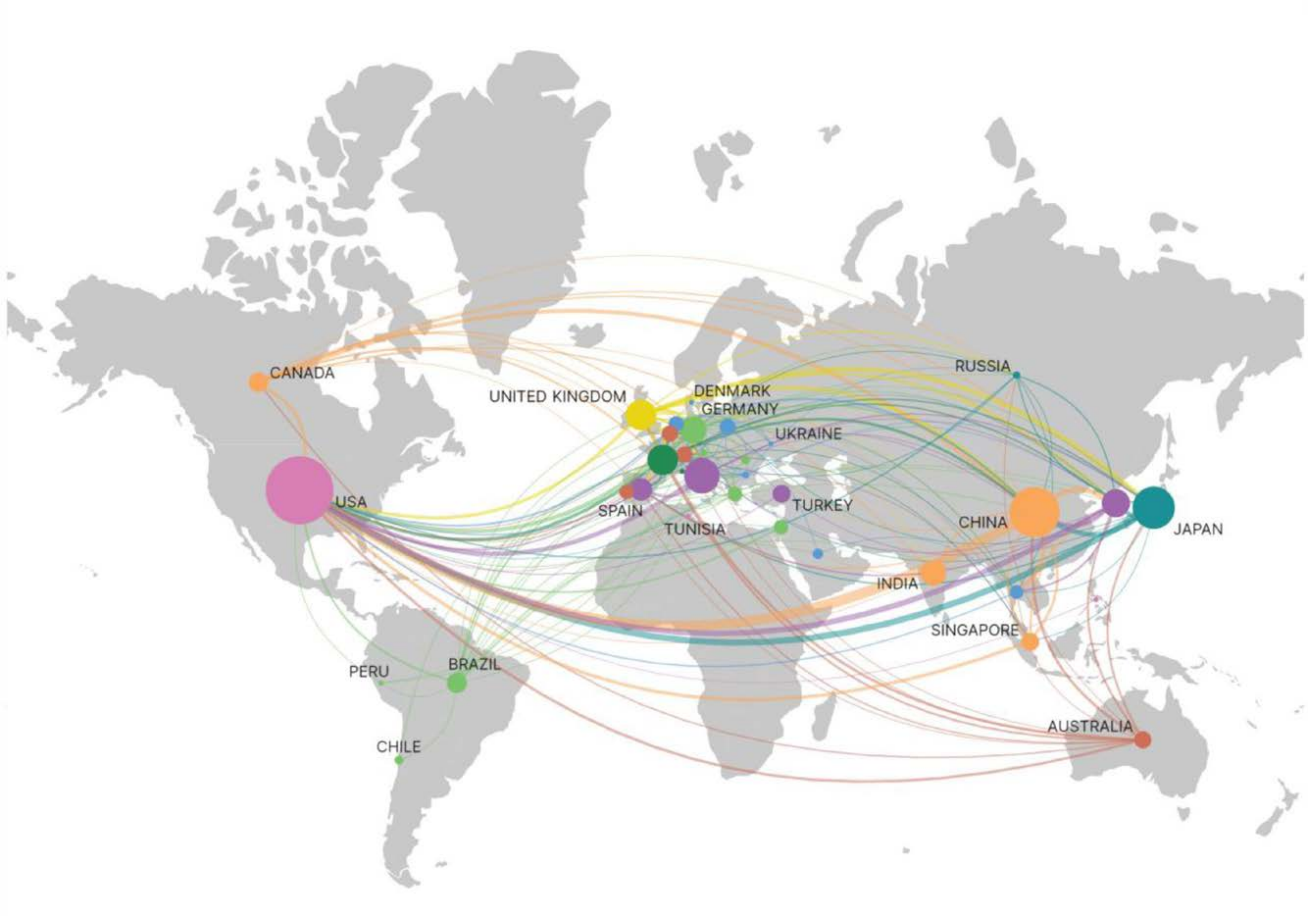
Geographical distribution and international networking of global publications in paronychia.

A total of 61 countries or regions worldwide have publications in the field of paronychia (Fig. 3). As shown in Table 1, the United States has the highest publication volume (n = 173), significantly surpassing the second-ranked Japan (n = 67) and the third-ranked China (n = 55). While the USA leads in total citations with 6433 records, however, it’s noteworthy that Germany (93.22), France (79.89), and England (72.84) have the highest citations per article. This highlights that papers from these regions warrant increased recognition. The network map reveals the USA as the epicenter of this research domain, with robust collaboration observed among different countries/regions (Fig. 3).

**Table 1:** The top 10 countries/regions making the most significant contributions to the field of paronychia-related research.

Rank	Country/Region	Publications	Citations	Citation per article
1	USA	173	6433	37.18
2	Japan	67	2367	35.33
3	China	55	2204	40.07
4	Italy	47	1659	35.30
5	Taiwan	39	2028	52.00
6	France	35	2796	79.89
7	England	31	2258	72.84
8	South Korea	30	1912	63.73
9	Germany	27	2517	93.22
10	India	24	419	17.46

### 3.3. Authors

**Figure 4.**
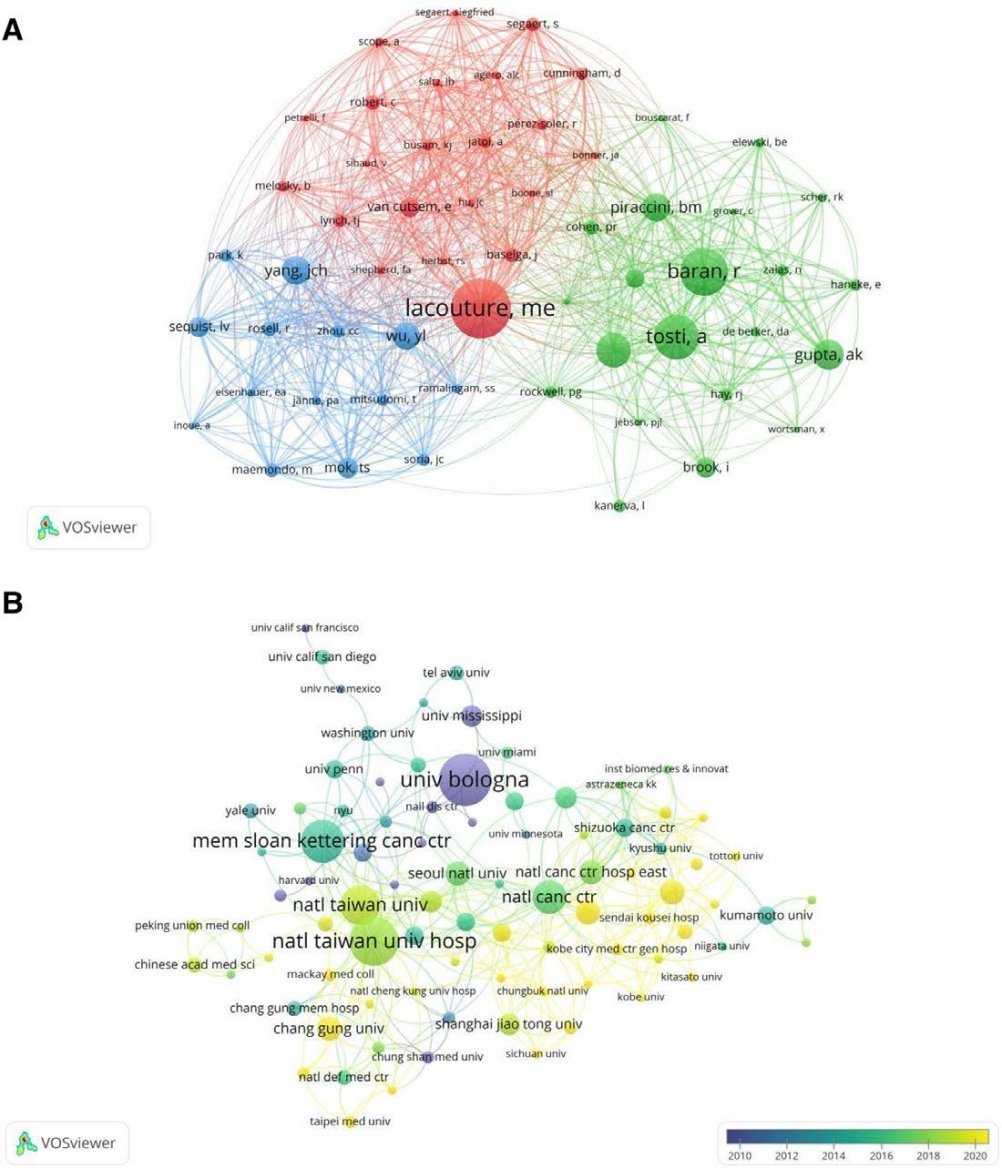
(A) Co-citation analysis of the first author in paronychia. (B) Co-authorship analysis of institutions in paronychia.

A total of 3035 authors contributed to paronychia. The authors with the highest publication volume in this field are Tosti A from Italy (n = 11), Lacouture ME from the United States (n = 10), and Piraccini BM from Italy (n = 7). The top 10 authors contribute to approximately 10.59% of the total publication volume. Notably, Lacouture ME (74.7), Tosti A (41.27), and Piraccin BM (39.57) have the highest average citation counts per article, indicating the substantial influence of these authors (Table 2). Figure 4A presents the co-citation analysis of the first author in the field of paronychia, the minimum citation count of an author was set as 20. Among these authors, Lacouture ME (1389), Baran R (1113), and Tosti A (1034) emerged as the top 3 authors in terms of total link strength.

**Table 2:** The top 10 most prolific authors in the domain of paronychia-related research.

Rank	Author	Country/Region	Publications	Citations	Citation per article
1	Tosti A	Italy	11	454	41.27
2	Lacouture ME	USA	10	747	74.70
3	Piraccini BM	Italy	7	277	39.57
4	Chu Chia-yu	Taiwan	6	109	18.17
5	Nakagawa K	Japan	6	104	17.33
6	Ahn Myung-ju	South Korea	5	97	19.40
7	Brook I	USA	5	123	24.60
8	Cohen PR	USA	5	190	38.00
9	Daniel CR	USA	4	95	23.75
10	Katakami N	Japan	4	84	21.00

### 3.4. Institutions

As shown in Table 3, the majority of the top 10 research institutions with the highest publication volume are from Asia, including 4 from Japan, 3 from Taiwan, and 1 each from South Korea, the United States, and Italy. The University of Bologna has the highest publication volume with 17 papers, followed by the National Taiwan University Hospital with 16 papers and the Memorial Sloan Kettering Cancer Center with 14 papers. The Memorial Sloan Kettering Cancer Center has the highest citation count (997 times), followed by the University of Bologna (622 times) and the National Cancer Center (386 times).The institutions with the highest average citation per paper are Memorial Sloan Kettering Cancer Center (71.21), University of Bologna (36.59), and National Cancer Center (35.09). Co-authorship of research institutions with a minimum of 3 publications was analyzed (Fig. 4B). Institutional cooperation in paronychia showed the National Taiwan University Hospital participated the most in collaborations with other institutions, with the highest total link strength (50), followed by National Cancer Center (31) and Kindai University (29).

**Table 3:** The leading 10 institutions in terms of publication volume within paronychia-related research.

Rank	Organization	Country/Region	Publications	Citations	Citation per article
1	University of Bologna	Italy	17	622	36.59
2	National Taiwan University Hospital	Taiwan	16	374	23.38
3	Memorial Sloan Kettering Cancer Center	USA	14	997	71.21
4	National Taiwan University	Taiwan	13	203	15.62
5	National Cancer Center	Japan	11	386	35.09
6	Kindai University	Japan	8	76	9.50
7	Seoul National University	South Korea	8	97	12.13
8	National Cancer Center Hospital East	Japan	8	120	15.00
9	National Hospital Organization	Japan	8	84	10.50
10	Chang Gung University	Taiwan	8	58	7.25

### 3.5. Journals

As shown in Table 4, the top 10 most productive journals are all from Journal Citation Reports (JCR) categories Q1 or Q2. For the regional distribution, 5 journals are from the United States, 2 from the England, and the remaining 3 comes from Switzerland, Netherlands, Germany. The journal with the highest publication volume is “Journal of the American Academy of Dermatology” (25), followed by “International Journal of Dermatology” (21), “Journal of the European Academy of Dermatology and Venereology” (12), and “Dermatology” (12). Additionally, “Journal of the American Academy of Dermatology” consistently ranks first in total citations, average citations per paper, impact factor, making it the most influential journal in the field of paronychia.

**Table 4:** The top 10 journals with the highest number of publications in the field of paronychia.

Rank	Journal	Country/Region	Publications	Citations	Citation per article	IF (2023)	JCR (2023)
1	Journal of the American Academy of Dermatology	USA	25	1276	51.04	13.8	Q1
2	International Journal of Dermatology	USA	21	540	25.71	3.6	Q2
3	Journal of the European Academy of Dermatology and Venereology	England	12	357	29.75	9.2	Q1
4	Dermatology	Switzerland	12	203	16.92	3.4	Q1
5	Supportive Care in Cancer	USA	11	402	36.55	3.1	Q2
6	Lung Cancer	Netherlands	11	185	16.82	5.3	Q1
7	Pediatric Dermatology	USA	11	79	7.18	1.5	Q2
8	Dermatologic Clinics	USA	10	180	18.00	2.4	Q1
9	Mycoses	Germany	10	266	26.60	4.9	Q1
10	British Journal of Dermatology	England	9	431	47.89	10.3	Q1

IF = impact factor, JCR = Journal Citation Reports.

**Figure 5.**
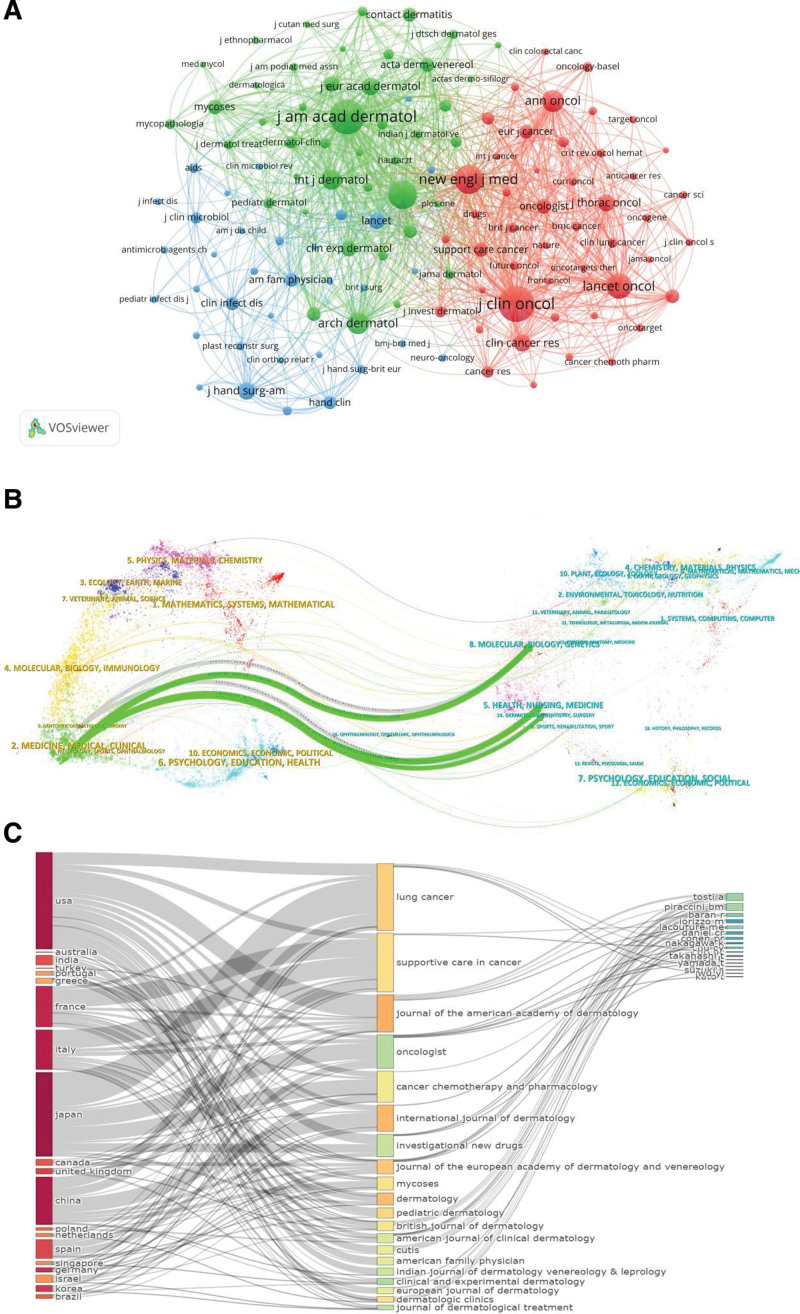
(A) Co-citation analysis of journals in paronychia. (B) Dual-map overlay of journals about paronychia. (C) The 3 fields plot.

Figure 5A shows the co-citation analysis of journals related to paronychia, with a minimum threshold of 20 citations for a single journal. Among 124 journals, the top 3 journals with the highest total link strength were Journal of the American Academy of Dermatology (24,085), Journal of Clinical Oncology (23864), and The New England Journal of Medicine (15,817). In the dual-map overlay of journals in Figure 5B, the left side of the curve represents the references cited in the study, while the right side of the curve indicates the cited reference source. Among 5 main citation paths, the green citation path with the highest z score in the field of “MEDICINE, MEDICAL, CLINICAL” mainly cited the literature published in the field of “MOLECULAR, BIOLOGY, GENETICS.” The 3-field plot reveals that high-producing countries typically contribute to literature published in journals with a high impact factor. Moreover, these journals demonstrate a strong association with core authors, as illustrated in Figure 5C.

### 3.6. Top references

**Figure 6.**
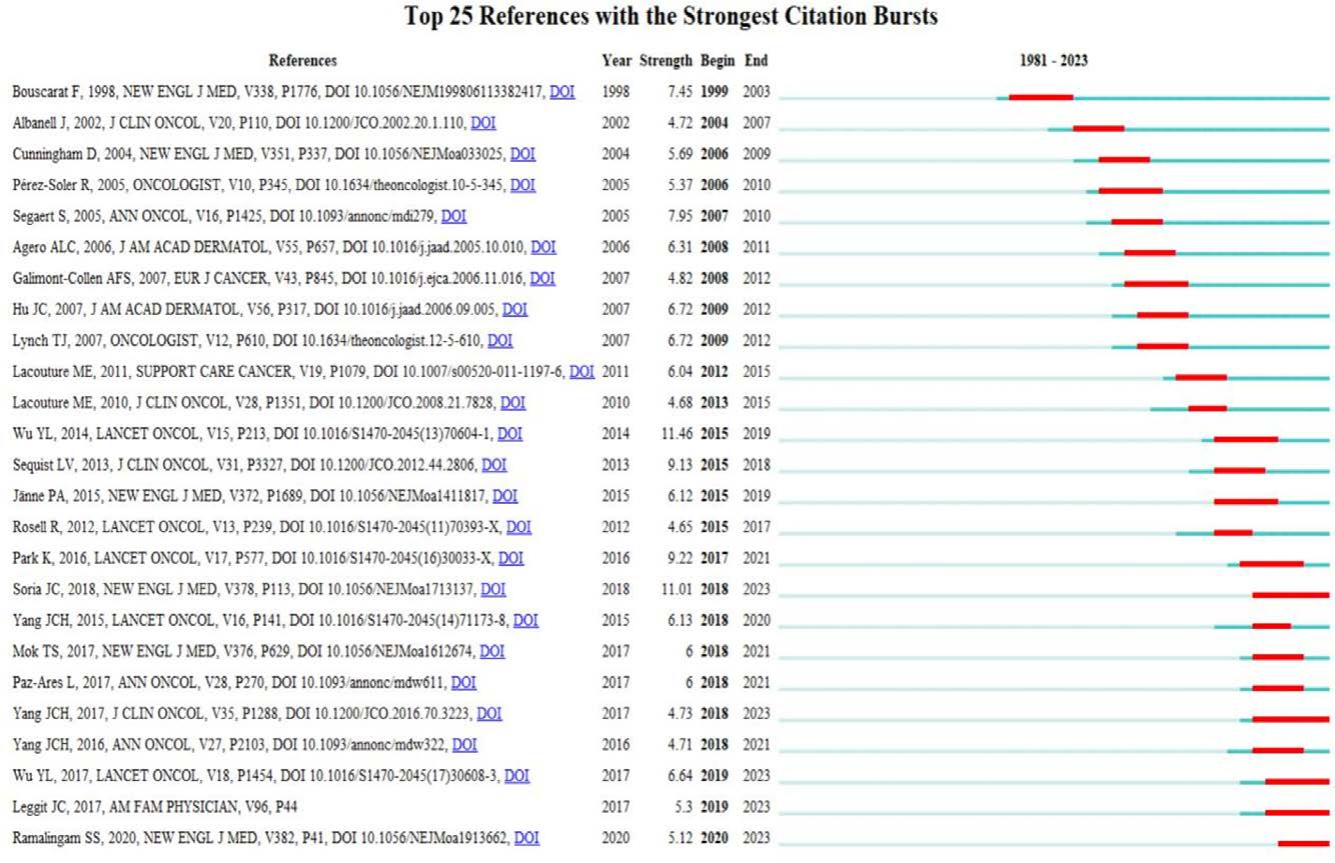
The top 25 references with the highest citation burst value.

We employed Citespace’s Burstness feature to identify papers that experienced a sudden surge in citations within a specific time frame (Fig. 6). It was found that the study conducted by Wu et al in 2014,^[18]^ titled “Afatinib versus cisplatin plus gemcitabine for first-line treatment of Asian patients with advanced non-small-cell lung cancer harboring EGFR mutations (LUX-Lung 6): an open-label, randomized phase 3 trial,” published in The Lancet Oncology, demonstrated the most robust citation burst (2015–2019, strength 11.46). They investigated the incidence rate of paronychia linked to the side effects of Afatinib in treatment of non-small-cell lung cancer. The research conducted by Soria et al in 2018,^[19]^ titled “Osimertinib in Untreated EGFR-Mutated Advanced Non-Small-Cell Lung Cancer,” boasts the strongest Citation Burst in recent 5 years (2018–2023, strength 11.01), their work analyzed the risk of paronychia while Osimertinib was applied in treatment of non-small-cell lung cancer.

### 3.7. Keywords

**Figure 7.**
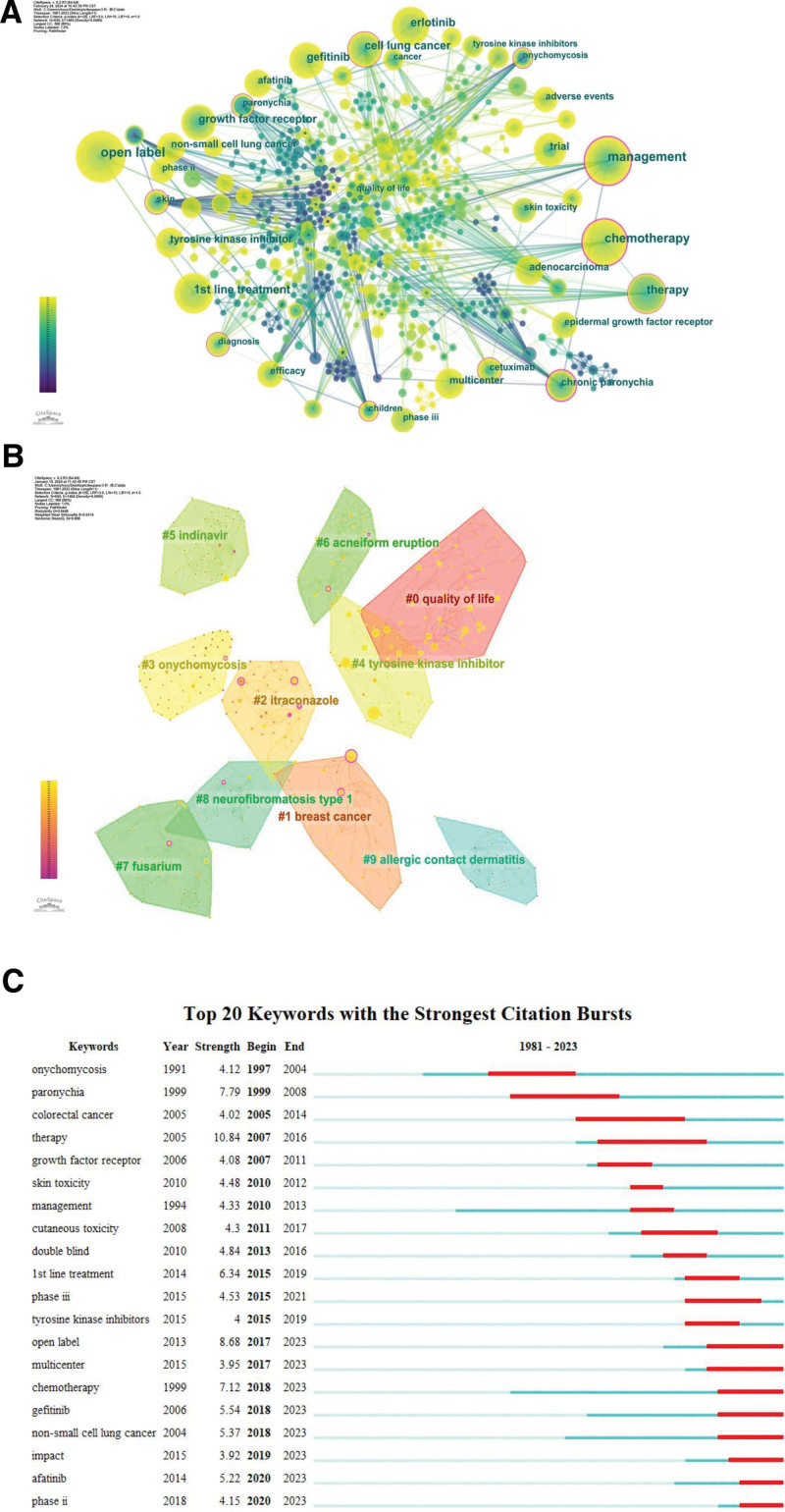
(A) The keyword co-occurrence map. (B) Keyword clustering, the clusters are sorted from 0 to 9 based on their size. (C) The top 20 keywords with the highest citation burst value.

In this work, keywords co-occurrence network was generated by Citespace (Fig. 7A). The “Years per slice” parameter was set as 1 year. Typically, the keywords appeared with a higher frequency mostly comes from much recent years, especially after 2010. While the keywords with centrality >0.1 are characterized by a purple outer ring, with the top 3 of them include “chronic paronychia” (centrality = 0.35), “management” (centrality = 0.21), “chemotherapy” (centrality = 0.20).

Through the likelihood ratio (LLR) algorithm, we conducted a keyword clustering analysis and identified the top 10 largest clusters (Fig. 7B). Notably, cluster 0 emerged as the largest, comprising 60 keywords. Table 5 presents comprehensive details regarding each cluster. All clusters exhibit a Silhouette value exceeding 0.8, indicating significant homogeneity within each cluster. Cluster 2, 3, 7, 8, and 9, all have a mean year predating 2007, which means studies within these clusters are relatively old, predominantly focused on topics about paronychia in etiology, epidemiology, bacteriology, diagnose,treatment, etc. Cluster 5 with a mean year 2003, most of the corresponding research related to human immunodeficiency virus-drug induced paronychia. Clusters emerging after 2009 include 0, 1, 4, and 6, featured by keywords associated with paronychia induced by the side effects anti-tumor drugs.

**Table 5:** Keyword clustering information based on the LLR algorithm of CiteSpace.

ClusterID	Size	Silhouette	Mean (yr)	Label (LLR)
#0	60	0.808	2015	Quality of life; acneiform rash; targeted therapy; skin toxicity; tyrosine kinase inhibitor
#1	53	0.897	2009	Breast cancer; adverse effects; antibiotic therapy; acute paronychia; pertuzumab
#2	49	0.939	2004	Itraconazole; chronic paronychia; fluconazole; infection; necrotizing fasciitis
#3	47	0.975	1997	Onychomycosis; dermatomycoses; epidemiology; infections; candida albicans
#4	42	0.891	2014	Tyrosine kinase inhibitor; non-small cell lung cancer; zd1839; phase i; afatinib
#5	42	0.957	2003	Indinavir; acitretin; adalimumab; ingrown toenails; acrodermatitis continua of hallopeau
#6	38	0.905	2013	Acneiform eruption; colorectal cancer; acne-like rash; radiation dermatitis; kras
#7	36	0.935	2007	Fusarium; occupational skin diseases; protein contact dermatitis; contact urticaria; diagnostik
#8	27	0.951	2003	Neurofibromatosis type 1; plexiform neurofibroma; emergence; children; clinical benefit
#9	25	0.992	1998	Allergic contact dermatitis; occupational; material safety data sheets; ethylene glycol dimethacrylate; resin additives

LLR = likelihood ratio.

In bibliometrics, the citation bursts of keywords signifies a period of intense research activity in a specific field. The top 20 keywords with the most strongest citation bursts are depicted in Figure 7C. Among them, the keyword with the highest burst intensity is “therapy.” Keywords with the longest sustained burst duration, spanning 9 years, include “paronychia,” “colorectal cancer,” and “therapy.” Notably, keywords such as “open label,” “multicenter,” “chemotherapy,” “gefitinib,” “non-small cell lung cancer,” “impact,” “afatinib,” and “phase ii” have sustained their burstiness from inception and persist to the present. Particularly, “open label” exhibits the most significant burst intensity in recent years.

## 4. Discussion

### 4.1. Historical changes in paronychia research

The historical evolution of paronychia research, as revealed by the bibliometric analysis, demonstrates a significant transformation in academic focus over time. Initially, there were limited publications, suggesting a gap in understanding or recognize paronychia as a significant health issue. However, starting from the 1990s, there has been a consistent growth in publication volume, reaching the peak in 2021. This surge may reflect an increased recognition of paronychia’s clinical importance, as well as the increasing newly emerged paronychia cases due to the EGFRI adverse events. The historical perspective underscores the dynamic nature of paronychia, which means it can not only appear as an independent condition^[2,20,21]^ but also may occur as a complication of other diseases^[22–24]^ or as a side effect during the course of drug therapies.^[25,26]^

### 4.2. Analysis of research entities

Research entities refer to the object that produces scientific outcomes, including countries/regions, authors, and institutions. Through a comprehensive bibliometric analysis of the research entities about paronychia, the global research landscape in this area was unveiled. The United States demonstrated exceptional prowess in paronychia research, exhibited leadership not only in the quantity of publications but also in international collaboration and research impact. Besides, countries such as Japan and China also made significant contributions to this field(Fig. 3A). Among the most highly productive authors, Tosti A from the University of Bologna explored the intricate relationship between paronychia and environmental factors, fungal infections, autoimmune blistering diseases, and antiretroviral drugs.^[9,27–29]^ In addition, Tosti A conducted further research on the treatment of paronychia.^[30]^ While the papers of Lacouture ME from the Memorial Sloan Kettering Cancer Center, ranked 2nd in publication volume, researched the risk of paronychia while using EGFRI in treatment of cancer.^[31,32]^ These outstanding researchers, with their remarkable productivity and citation impact, play a pivotal role in advancing both the diagnosis and treatment of paronychia. Analysis of the collaborative network reveals that most countries, institutions, and authors formed closely-knit collaborative groups. This multinational collaboration facilitates resource sharing, knowledge exchange, and diverse research perspectives. It lays a solid foundation for future collaborative research efforts, poised to propel in-depth and comprehensive advancements in paronychia-related research.

### 4.3. Etiology, clinical manifestations, and treatment advances

The cause of paronychia are multiple,which include excessive nail trimming, improper footwear, ingrown nails, and prolonged exposure to chemical substances, etc. Sometimes, it may appear as a complication of pemphigus vulgaris or aggressive digital papillary adenocarcinoma as well. The fundamental pathogenesis involves the disruption of the skin barrier around the nail, which lead to microbial invasion and localized infections.

Paronychia is primarily caused by pathogens such as Staphylococcus aureus, Enterococcus faecalis, gamma-hemolytic streptococci and Enterobacter cloacae, virus, fungi, etc.^[23,33,34]^ These bacteria may exist on the skin of healthy individuals without causing infections. In the early stages of infection, there may be slight redness and swelling of the soft tissues around the nail bed, accompanied by a burning or swelling sensation at the fingertip or nail groove, along with mild to moderate pain.^[35]^ As the condition worsens, there might be abscess formation at the surrounding soft tissues of nail, which may lead to local skin ulceration with the discharge of pus.^[2]^ In some cases, due to the accumulation of pus under the nail plate or the potent toxicity of the pathogenic bacteria, the infection may further spread to the finger bone, causing suppurative osteoarthritis or even systemic inflammatory reactions such as fever and sepsis.^[3,36]^ In the early stages of purulent paronychia, symptoms of most cases can effectively controlled through conservative treatments, including maintaining hand hygiene, targeted application of antibiotics, and pain relievers.^[2,37,38]^ While patients with evident localized abscess formation, surgical treatment for abscess drainage are usually necessary.^[20]^ The primary purpose of surgery is to promptly clear the infection and prevent it spread further. In recent years, El-Komy et al^[39]^ explored the application of laser in managing paronychia, Liu et al^[40]^ tried to treat paronychia with a Ω Toenail Correction scaffold, both reduced the risk of surgical intervention and relived the pain. In conclusion, less invasive treatment approach can be the main direction in the future.

### 4.4. Risks and treatments associated with EGFR inhibitor-induced paronychia

In recent years, the drug-related paronychia are relatively common due to the advancements of the pharmaceutical industry. Among them, the EGFRI-induced paronychia (Gefitinib, Erlotinib, Afatinib, etc.) was the most reported type of drug-related paronychia. Currently, it has been confirmed that the high concentrations of drugs in the local tissues of fingers and toes are not associated with the onset of paronychia,^[41]^ and the exact mechanisms remain unclear. Furthermore, the incidence of paronychia varies among different anti-cancer drugs. Studies by Wang et al^[12]^ reported a 16.2% incidence with Gefitinib, Wu et al^[42]^ found a 20.8% incidence with Erlotinib, and Cheema et al^[13]^ identified a 45.5% incidence with Afatinib. Paronychia, as a side effect of anti-cancer drugs, is prone to secondary bacterial infections.. Nevertheless, the efficacy of antibiotic treatment is limited, and symptoms can be effectively ameliorated by local application of corticosteroids or by reducing the dosage of EGFRI.^[25]^

### 4.5. Research trends and future directions

In terms of the keywords burst, chemotherapy, afatinib, gefitinib, and non-small cell lung cancer are the most representative keywords persisting in recent years. This suggests that subsequent research hotspots in this area are likely to be EGFRI related paronychia. Based on the current background, it implies the subsequent research should focus more on differentiating and treating for paronychia induced by newer generations of EGFRI drugs.

## 5. Limitations

Our research offers an overview of the present research status in paronychia, accompanied by visual mappings, trend analyses, and identification of focal areas, thus furnishing a roadmap for following inquiries. Nonetheless, our study faces several constraints. Primarily, data extraction was restricted to WoSCC, neglecting other pertinent databases such as Medline and Embase. Additionally, solely English-language publications were incorporated, limited the scope.

## 6. Conclusion

This comprehensive bibliometric analysis illuminates the dynamic landscape of paronychia research, revealed that most of the recent hotspots are not focused on the basic research about paronychia. This situation may arise from the fact that research on the pathogenesis, microbiological characteristics, and treatment strategies of traditional type paronychia has reached a mature stage, making significant breakthroughs can be challenging. The collaborative efforts among countries/regions, institutions, and authors, notably spearheaded by the United States, fostered a tightly-knit international cooperation network. Keyword-based clustering analysis suggests that the research hotspot on paronychia in the past 2 decades has consistently focused on the paronychia induced by EGFRI. This is associated with the widespread clinical application of EGFRI-class drugs, leading to a significant increase in the unavoidable number of drug-induced paronychia patients. Therefore, doctors need to pay attention to whether patients have a history of EGFRI medication when diagnosing paronychia. With the ongoing advancement of EGFRI-class drugs and the gradual adoption of newer generation medications, this field calls for increased international collaboration and deeper research efforts. Meanwhile, treatment options with less pains and trauma equally deserve further research attention. In light of the expanding diversity in the etiology of paronychia, advancing clinical developments in this field necessitate collaborative endeavors among dermatologists, orthopedists, emergency physicians, oncologists, pharmacists, and professionals from various other disciplines.

## Acknowledgments

We would like to express our heartfelt thanks to Lelanie Tabanao Lubao for her assistance in polishing the paper.

## Author contributions

**Conceptualization:** Chaoxi Zhou.

**Data curation:** Zhaoyi Yang, Huimin Wang, Yongzhen Zhao.

**Formal analysis:** Chaoxi Zhou.

**Funding acquisition:** Huimin Wang.

**Methodology:** Qinglei Wang.

**Software:** Guangrong Yu.

**Supervision:** Qinglei Wang.

**Visualization:** Guangrong Yu.

**Writing – original draft:** Chaoxi Zhou.

**Writing – review & editing:** Chaoxi Zhou.

## Supplementary Material


